# Knowledge, Attitudes, and Practices toward COVID-19 and Vaccines among Chinese Small-Town Residents: A Cross-sectional Study

**DOI:** 10.4269/ajtmh.22-0031

**Published:** 2022-07-25

**Authors:** Si-Yi Yu, Jun-Jun Luo, Hong-Yao Cui, Ke-Shu Shan, Lei Xu, Ling Ding, Xue-Qin Chen

**Affiliations:** ^1^ Department of General Internal Medicine, Ningbo First Hospital, Ningbo, Zhejiang, China;; ^2^ Department of Nursing, Ningbo First Hospital, Ningbo, Zhejiang, China;; ^3^ Department of General Internal Medicine, Ningbo Haishu No. 2 Hospital, Ningbo, Zhejiang, China;; ^4^ Department of Traditional Medicine, Ningbo First Hospital, Ningbo, Zhejiang, China

## Abstract

China has basically controlled the COVID-19 epidemic as a result of widespread vaccination and other containment strategies, despite localized outbreaks, as of September 2021. This study investigates the knowledge, attitudes, and practices (KAP) of COVID-19 and COVID-19 vaccines among Chinese small-town residents to provide suggestions for public health policy. Residents who were vaccinated against COVID-19 were asked to complete a paper questionnaire on KAP in Xidian, Zhejiang. The knowledge questionnaire consisted of 12 questions regarding COVID-19 and 12 questions regarding COVID-19 vaccines. Attitude and practice evaluation included agreement on the eventual control of COVID-19 and having recently worn a mask outside. Of 405 survey responders, 52.3% were male, 71.4% had middle school education or less, and 59.0% engaged in physical labor as an occupation. The correct answer rates of the COVID-19 section and the vaccine section were 79.2% and 71.7%, respectively. Age groups of 18 to 29 years and > 50 years, occupations of physical labor and unemployment, and primary school education and less were associated with lower knowledge scores. The majority of participants (91.6%) believed that COVID-19 will eventually be controlled, whereas women, students, and patients with chronic held relatively negative attitudes toward epidemic control. Most participants (87.4%) wore masks outside recently. In conclusion, Chinese small-town residents have a medium level of knowledge regarding COVID-19 and vaccines, hold positive attitudes, and have appropriate practices. Health education should be provided to the target populations to enrich their knowledge of COVID-19 and vaccines, and to improve their attitudes toward epidemic control.

## INTRODUCTION

COVID-19 is a respiratory disease caused by a novel coronavirus named severe acute respiratory syndrome coronavirus 2. The virus shows various transmission routes and strong interpersonal infectivity, spreading rapidly through respiratory droplets and direct contact.^1^ On January 30, 2020, the WHO declared the COVID-19 outbreak a public health emergency of international concern, calling on all countries to take urgent action to prevent the pandemic.^2^ By September 24, 2021, the disease had resulted in 230,418,451 infections and 4,724,876 deaths.^3^

To relieve the burden of COVID-19 and the depletion of medical resources, effective and safe vaccines are needed desperately. Currently, messenger RNA vaccines, adenovirus vaccines, and inactivated virus vaccines have been developed and are being administered.^4^ China has promoted COVID-19 vaccination extensively, which has been free for all people older than 18 years since January 2021.^5^ A total of 2,174,043,000 doses of COVID-19 vaccines were administered in China by September 19, 2021.^6^

As of September 2021, China is one of the countries that has better managed the COVID-19 epidemic as a result of strict control measures and widespread vaccination.^7^ Nonetheless, people’s knowledge of COVID-19 and vaccines may affect their adherence to public health measures.^8^ A Chinese online study at the initial phase of the epidemic showed that residents of a high socioeconomic status held good knowledge of COVID-19 and had positive attitudes and proper practices.^9^ Another online survey from China revealed that residents from rural areas had a medium level of knowledge and positive attitudes toward COVID-19,^10^ but the online sample might lack representativeness because of poor Internet connectivity and accessibility in rural populations. To date, few offline studies have been concerned about the knowledge, attitudes, and practices (KAP) of COVID-19 and vaccines among less-educated people and physical workers.

Xidian town, Zhejiang Province is a small town dominated by an industrial economy on the southeast coast of China. The total population is approximately 100,000, including 55,000 immigrants (mainly migrant workers) with a relatively low education level.^11^ To facilitate the control of the COVID-19 epidemic in the postvaccination era, we conducted a paper questionnaire to survey residents in Xidian who were vaccinated against COVID-19, on their KAP of COVID-19 and the vaccine.

## METHODS

### Participants.

This cross-sectional study was conducted at the Xidian vaccination site between August 18, 2021 and September 8, 2021. Residents vaccinated against COVID-19 were recruited, whereas those who were younger than 18 years, unable to understand the study, or unwilling to participate were excluded.

### Measures.

Paper questionnaires were distributed to the participants during the observation period after the COVID-19 vaccination. For those who had difficulty filling out questionnaires for various reasons, such as visual impairment and low education level, the surveyors helped read the contents and fill in the questionnaire. The questionnaire was developed by the research team and consisted of three sections: demographics, knowledge, and attitudes/practices. Demographic variables included age, gender, marital status, place of registration, education, occupation, and medical history.

The knowledge assessment included two sections: knowledge of COVID-19 and knowledge of COVID-19 vaccines (Supplemental Table S1). Knowledge of COVID-19 referred to a previous study^9^ and was excerpted from *the Guidelines for the Diagnosis and Treatment of Coronavirus Disease 2019 (Trial Version 8)* and the revised version published by the National Health Commission of the People’s Republic of China.^12^^,^^13^ This section had 12 questions: four regarding clinical manifestations (K1.1–K1.4), three regarding transmission routes (K1.5–K1.7), and five regarding the prevention and control of COVID-19 (K1.8–K1.12). Knowledge of COVID-19 vaccines referred to *Guidelines of Vaccination for COVID-19 Vaccines in China (1st Edition)* published by the National Health Commission of the People’s Republic of China.^14^ This section also had 12 questions: one regarding COVID-19 vaccination policy in China (K2.1), seven regarding indications and contraindications for the vaccine (K2.2–K2.8),^15^ one regarding vaccine adverse reactions (K2.10), two regarding vaccine effectiveness (K2.9 and K2.12), and one regarding postvaccination prevention and control measures (K2.11). The options for answers included True, False, and I don’t know. A correct answer was scored 1 point; an incorrect/unknown answer was scored 0 point. The total score of each section was between 0 and 12 points, and a higher score represented better knowledge. The Cronbach’s alpha coefficient of the knowledge section was 0.756, indicating acceptable internal consistency.^16^

Attitudes and practices referred to a previous study.^9^ Attitudes toward COVID-19 were evaluated by two questions (A1 and A2, Supplemental Table S1), and practices were assessed by two questions (P1 and P2, Supplemental Table 1).

Because of the large population, the sample size was based on the formula $n\geq {\left(\frac{k}{\alpha }\right)}^{2}P(1-P).$^17^ When *α* = 0.05, *k* = 1.96, and *P* = 0.5, the sample size was 384. Assuming the loss to follow-up rate was 10%, the estimated sample size was 427.

### Statistical analysis.

Data were analyzed using IBM SPSS (version 26.0; SPSS Inc., Chicago, IL). Continuous variables were presented as the mean ± SD. Independent sample *t-*tests, one-way analysis of variance, or χ^2^ tests were adopted to compare the knowledge scores, attitudes, and practices of different groups when appropriate. Multiple linear regression analysis was applied to identify the demographic factors associated with the knowledge score, and binary logistic regression analysis was used to identify the factors related to attitudes and practices. A backward stepwise method was used to select factors. The nonstandard regression coefficient (β), odds ratio (OR), and the 95% CI was used to represent the correlation between variables and KAP. *P* < 0.05 was considered statistically significant.

## RESULTS

**Figure 1.**
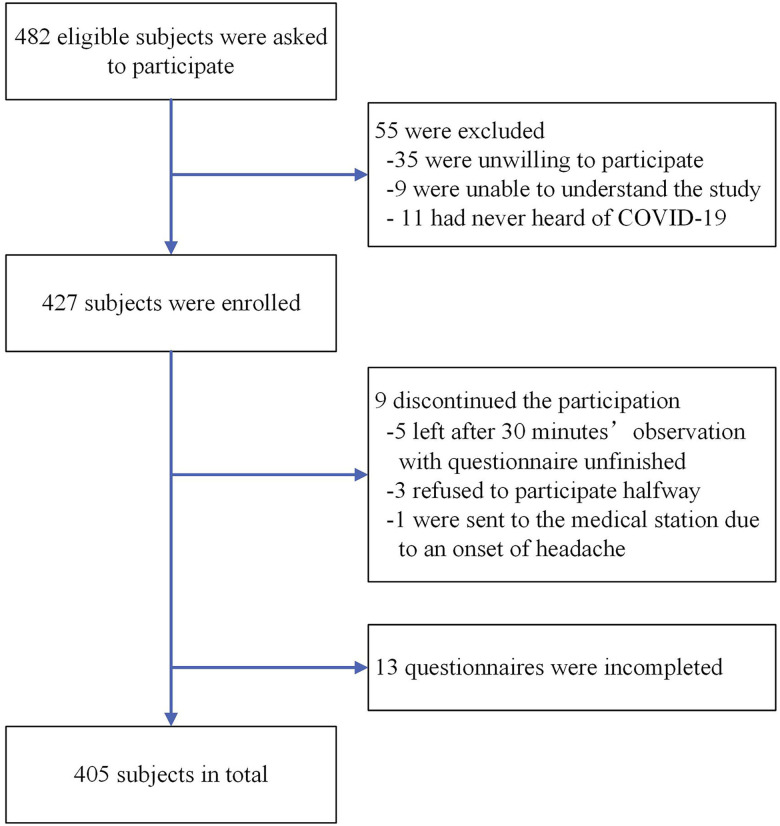
Inclusion flow chart.

The inclusion flow chart is shown in Figure 1. A total of 482 eligible residents were asked to participate in the survey, 55 of whom were excluded according to the exclusion criteria (35 were unwilling to participate, 9 were unable to understand the study, and 11 had never heard of COVID-19). A total of 427 participants were asked to fill in the paper questionnaire, all of which were retrieved. Excluding nine subjects who suspended the study (five left after the observation time, three declined to participate halfway through, and one went to the medical center because of an onset of headache) and 13 subjects with incomplete questionnaires, a total of 405 participants were finally recruited. As shown in Table 1, the average age was 43.6 years (SD, 15.3 years; range, 18–84 years), 212 (52.3%) were male, 289 (71.4%) had a middle school education or less, 239 (59.0%) engaged in physical labor as an occupation, and 217 (53.6%) immigrated from populations outside Zhejiang Province.

**Table 1:** Knowledge score of COVID-19 and vaccines by demographic variable

Variable	No. of participants (%)	Knowledge score of COVID, mean ± SD	*P *value	Knowledge score of vaccines, mean ± SD	*P *value	Aggregate knowledge score, mean ± SD	*P *value
Gender							
Male	212 (52.3)	9.73 ± 1.80	0.009	8.77 ± 1.72	0.062	18.50 ± 2.88	0.007
Female	193 (47.7)	9.24 ± 1.91		8.44 ± 1.81		17.68 ± 3.20	
Age, y							
18–29	88 (21.7)	9.72 ± 1.55	< 0.001	8.41 ± 1.73	< 0.001	18.13 ± 2.64	< 0.001
30–49	164 (40.5)	10.22 ± 1.51		9.04 ± 1.67		19.26 ± 2.70	
> 50	153 (37.8)	8.60 ± 2.02		8.27 ± 1.81		16.88 ± 3.18	
Marital status							
Married	334 (82.5)	9.44 ± 1.97	0.285	8.68 ± 1.80	0.093	18.12 ± 3.19	0.428
Never married	67 (16.5)	9.73 ± 1.27		8.22 ± 1.57		17.96 ± 2.29	
Divorced or widowed	4 (1.0)	10.50 ± 1.29		9.50 ± 1.73		20.00 ± 2.94	
Education							
Primary school or less	132 (32.6)	8.40 ± 2.04	< 0.001	8.14 ± 1.81	< 0.001	16.54 ± 3.25	< 0.001
Middle school	157 (38.8)	9.81 ± 1.64		8.96 ± 1.80		18.77 ± 2.86	
High school	82 (20.2)	10.17 ± 1.27		8.52 ± 1.60		18.70 ± 2.38	
Bachelor’s degree or more	34 (8.4)	10.71 ± 1.27		9.06 ± 1.32		19.76 ± 2.09	
Occupation							
Physical labor	239 (59.0)	9.44 ± 1.86	< 0.001	8.68 ± 1.81	0.003	18.12 ± 3.07	< 0.001
Mental labor	69 (17.0)	10.55 ± 1.08		9.09 ± 1.40		19.64 ± 2.05	
Student	11 (2.7)	10.45 ± 1.13		7.64 ± 1.57		18.09 ± 2.12	
Unemployed	86 (21.2)	8.70 ± 2.04		8.17 ± 1.80		16.87 ± 3.27	
Medical history							
Yes	73 (18)	8.60 ± 1.93	< 0.001	8.30 ± 1.84	0.097	16.90 ± 3.11	< 0.001
No	332 (82)	9.70 ± 1.80		8.68 ± 1.75		18.38 ± 2.99	

The average aggregate score of the knowledge questionnaire was 18.1 points (SD, 3.1 points; range, 7–24 points), and the overall correct rate was 75.4% (18.1/24 × 100). The correct rate of 12 questions on COVID-19 knowledge was 30.9% to 98.8%. The average score was 9.5 points (SD, 1.9 points; range, 4–12 points), and the overall correct rate was 79.2% (9.5/12 × 100). The correct rate of 12 questions about vaccine knowledge was 10.9% to 98%. The average score was 8.6 points (SD, 1.8 points; range, 3–12 points), and the overall correct rate was 71.7% (8.6/12 × 100) (Supplemental Table S1). Knowledge scores revealed significant differences across genders, age groups, education levels, occupations, and disease status (*P* < 0.05; Table 1). Multiple linear regression analysis showed that the age groups of 18 to 29 years (β = –1.355, *P* < 0.001) and > 50 years (β = –1.353, *P* < 0.001) (versus 30–49 years), primary school education and less (versus a bachelor’s degree and more; β = –1.715, *P* < 0.001), and occupation of unemployed (versus mental labor; β = –1.096, *P* = 0.022) were associated significantly with lower aggregate knowledge scores. Similarly, age groups of 18 to 29 years (β = –0.651, *P* = 0.003) and > 50 years (β = –0.945, *P* < 0.001) (versus 30–49 years old), primary school education and less (versus bachelor’s degree and more; β = –1.095, *P* < 0.001), occupations of physical laborer (*β* = –0.633, *P* = 0.004) and unemployed (β = –0.831, *P* = 0.003) (versus mental labor) correlated significantly with lower COVID-19 knowledge scores. Age groups of 18 to 29 years (β = –0.673, *P* = 0.004) and >50 years (β = –0.502, *P* = 0.020) (versus 30–49 years), primary school education and less (versus bachelor’s degree and more; β = –0.753, *P* = 0.001) correlated significantly with a lower knowledge score of COVID-19 vaccines (Supplemental Table S2).

The majority of participants believed that COVID-19 would eventually be controlled successfully (91.6%). The proportions of responses of “disagree” and “I don’t know” were 2.0% and 6.4%, respectively. People’s knowledge scores (including COVID-19 knowledge as well as vaccine knowledge) in descending order were those who answered “disagree,” “agree,” and “I don’t know,” but with no significant difference. Because the analysis of variance failed to identify any influencing factor among the three types of responses, we combined “disagree” and “I don’t know” into the same category. Attitudes toward final success in epidemic control differed significantly across genders, occupations, and disease status (*P* < 0.05; Table 2). Binary logistic regression analysis showed that females (versus males; OR, 0.40; *P* = 0.024), occupation of student (versus mental labor; OR, 0.13; *P* = 0.010), and chronic patients (versus nonpatients; OR, 0.32; *P* = 0.010) tended to answer “disagree” or “I don’t know” on A1 (Supplemental Table 3).

**Table 2:** Attitude toward COVID-19 by demographic variable

Variable	Attitude of final success in controlling, n (%) or mean (SD)		
	Agree	Disagree	Don’t know
Gender, *n *(%)			
Male	201 (94.8)	3 (1.4)	8 (3.8)
Female	170 (88.1)	5 (2.6)	18 (9.3)*
Age, y; *n *(%)			
18–29	80 (90.9)	1 (1.1)	7 (8.0)
30–49	153 (93.3)	4 (2.4)	7 (4.3)
> 50	138 (90.2)	3 (2.0)	12 (7.8)
Marital status, *n *(%)			
Married	306 (91.6)	6 (1.8)	22 (6.6)
Never-married	61 (91.0)	2 (3.0)	4 (6.0)
Divorced or widowed	4 (100)	0 (0)	0 (0)
Education, *n *(%)			
Primary school or below	118 (89.4)	3 (2.3)	11 (8.3)
Middle school	150 (95.5)	1 (0.6)	6 (3.8)
High school	74 (90.2)	2 (2.4)	6 (7.3)
Bachelor’s degree or more	29 (85.3)	2 (5.9)	3 (8.8)
Occupation, *n *(%)			
Physical labor	221 (92.5)	4 (1.7)	14 (5.9)
Mental labor	64 (92.8)	1 (1.4)	4 (5.8)
Student	7 (63.6)	1 (9.1)	3 (27.3)
Unemployed	79 (91.9)	2 (2.3)	5 (5.8)†
Medical history, *n *(%)			
Yes	62 (84.9)	0 (0)	11 (15.1)
No	309 (93.1)	8 (2.4)	15 (4.5)*
Knowledge score of COVID, mean (SD)	9.51 (1.84)	10.63 (1.60)	9.04 (2.24)
Knowledge score of vaccines, mean (SD)	8.65 (1.76)	8.88 (1.13)	8.00 (1.96)
Aggregate knowledge score, mean (SD)	18.16 (3.02)	19.50 (2.39)	17.04 (3.59)

* *P* < 0.05.

† *P* < 0.01.

Nearly all participants (99.8%) had confidence that China can defeat COVID-19, whereas only one person (0.2%) was uncertain about the answer. We did not conduct influencing factor analysis in this part.

In recent days, most responders reported not having been to any crowded places (71.4%) and they wore a mask outside (87.4%). A small portion of participants still visited crowded places (28.4%) and left home without a mask (12.6%). The rate of visiting crowded places differed across age, occupation, and disease status (*P* < 0.05), and the rate of not wearing a mask differed across age, education level, and knowledge score (*P* < 0.05). Furthermore, binary logistic regression analysis showed that residents who were physical laborers (versus mental laborers; OR, 0.45; *P* = 0.010) tended not to visit crowded places. Gender, age, occupation, education level, disease status, and knowledge score failed to show any correlation with not wearing a mask outside (*P* > 0.05; Table 3).

**Table 3:** Practices toward COVID-19 by demographic variable

Variable	Practices			
	P1: going to a crowded place		P2: wearing a mask	
	Yes	No	Yes	No
Gender, *n *(%)				
Male	53 (25.0)	159 (75.0)	185 (87.3)	27 (12.7)
Female	63 (32.6)	130 (67.4)	169 (87.6)	24 (12.4)
Age, y; *n *(%)				
18–29	23 (26.1)	65 (73.9)	81 (92.0)	7 (8.0)
30–49	38 (23.2)	126 (76.8)	148 (90.2)	16 (9.8)
> 50	55 (35.9)	98 (64.1)*	125 (81.7)	28 (18.3)*
Marital status, *n *(%)				
Married	96 (28.7)	238 (71.3)	291 (87.1)	43 (12.9)
Never married	17 (25.4)	50 (74.6)	60 (89.6)	7 (10.4)
Divorced or widowed	3 (75)	1 (25)	3 (75)	1 (25)
Education, *n *(%)				
Primary school or less	40 (30.3)	92 (69.7)	104 (78.8)	28 (21.2)
Middle school	40 (25.5)	117 (74.5)	142 (90.4)	15 (9.6)
High school	23 (28.0)	59 (72.0)	77 (93.9)	5 (6.1)
Bachelor’s degree or more	13 (38.2)	21 (61.8)	31 (91.2)	3 (8.8)†
Occupation, *n *(%)				
Physical labor	54 (22.6)	185 (77.4)	202 (84.5)	37 (15.5)
Mental labor	25 (36.2)	44 (63.8)	65 (94.2)	4 (5.8)
Student	4 (36.4)	7 (63.6)	9 (81.8)	2 (18.2)
Unemployed	33 (38.4)	53 (61.6)*	78 (90.7)	8 (9.3)
Medical history, *n *(%)				
Yes	30 (41.1)	43 (58.9)	59 (80.8)	14 (19.2)
No	86 (25.9)	246 (74.1)†	295 (88.9)	37 (11.1)
Knowledge score of COVID, mean (SD)	9.54 (1.92)	9.48 (1.85)	9.60 (1.82)	8.76 (2.04)†
Knowledge score of vaccines, mean (SD)	8.60 (1.67)	8.62 (1.81)	8.68 (1.73)	8.14 (1.96)*
Aggregate knowledge score, mean (SD)	18.15 (3.14)	18.10 (3.03)	18.29 (3.00)	16.90 (3.22)†

* *P* < 0.05.

† *P* < 0.01.

## DISCUSSION

As far as we are concerned, this is the first offline research to investigate the KAP toward COVID-19 and COVID-19 vaccines among Chinese small-town residents. In this population with a relatively low education and a predominant occupation of physical laborer, the correct rate of overall knowledge questionnaire was 75.4%, with the COVID-19 section being 79.2% and the vaccine section being 71.7%. These data indicate that most participants had a median level of knowledge about COVID-19 and vaccines.

The majority of responders were optimistic toward the control of the COVID-19 epidemic; 91.6% of the study sample agreed about the final control of COVID-19, and 99.8% believed China could defeat the virus. Even so, the behavior of Chinese small-town residents is still cautious. In a time of widespread vaccination and a basically controlled epidemic, most people avoided crowded places (71.4%) and wore masks when going out (87.4%).

The correct rate of COVID-19 knowledge among small-town residents was 79.2%, which was less than a previous study by Zhong et al.^9^ We consider this was a result of different sample characteristics. Our participants were small-town residents, 71.4% of whom possessed a middle school education or less and 59.0% were engaged in physical labor. Zhong et al.^9^ focused on a population with a relatively high socioeconomic level. Although there is various information of COVID-19 and vaccines on the website of the National Health Commission of China and other health websites, residents with lower education levels and physical laborers may seldom acquire knowledge via these channels. The close correlation between education level/occupation and the COVID-19 knowledge score in our study and previous data supports this speculation.^9^^,^^18^^–^^20^ The correct rate of COVID-19 vaccine knowledge was 71.7%, and poorer knowledge was associated with a lower education level, similar to a study from Oman.^21^ These findings provide evidence for public health policymakers to identify target populations for COVID-19 education. Specific health education toward populations with poorer knowledge (age group of > 50 years, primary school education or less, physical laborer or unemployed) will work more efficiently. Moreover, targeted health education methods using some theory such as a health beliefs model may assist the effectiveness of health promotion.

The agreement about the final success and confidence in defeating COVID-19 was similar to that of a previous study done in the early stage of the epidemic.^9^ Although the worldwide pandemic had lasted for more than 21 months, Chinese residents still held positive attitudes on controlling the epidemic. This may be attributed to China’s effective control measures and universal vaccination policy. Despite small outbreaks in Zhengzhou, Nanjing, Fujian, and other places in recent months, local governments have quelled the outbreak in a short time via effective actions, including control of infectious sources, isolation and treatment of patients, and screening of close contacts. The quick and efficient measures strengthened residents’ confidence in defeating the virus.

Despite optimistic attitudes on COVID-19 control, most residents adopted preventive measures: avoided visiting crowded places and wore masks when leaving home. This was mainly a result of strict prevention and control measures implemented by the government, such as mandatory mask policies in public places, including markets, supermarkets, and public transportation. In addition, this practice was associated with residents’ good knowledge of the transmission routes of the virus and preventative measures. However, 28.4% of residents still visited crowded places, and 12.6% had not recently worn a mask outside. These rates were higher than previous data^9^ for the probable reason that people have let their guard down and partly returned to their habits by reasoning that the epidemic was basically controlled. Physical laborers tended not to visit crowded places, which might relate to their job specification.

Unlike previous studies,^9^^,^^17^ the knowledge level of residents in our study was not firmly associated with their attitudes and practices except for wearing masks outside. The reason might be that people were generally optimistic about epidemic control and had partially returned to their habit of visiting public places within the condition of a basically controlled epidemic in China. These factors might weaken the influence of knowledge level on attitudes and practices. This dilemma—success breeds complacency—is noted globally. For example, younger parents did not vaccinate their children for diseases when childhood vaccination rates were high; people stopped seeking care for fevers when malaria was close to elimination. Health authorities need to plan for this inevitable consequence of success, and more nuanced health promotion messages are required to sustain protective public health measures by individuals and households.

Our research has two strengths. First, we focus on the KAP of COVID-19 and vaccines among small-town residents in China, which has not been taken into consideration previously.^9^ Small-town residents comprise a considerable proportion of the Chinese population, and their KAP for COVID-19 and vaccines can influence epidemic prevention and control. In our study, subjects with a low education level, occupation of physical labor, and age group of > 50 years accounted for a large proportion of respondents. They might have limited acceptance of health knowledge from the media and less access to online health resources, which results in a lack of knowledge of COVID-19 and vaccines, and even negative attitudes. Second, unlike previous online surveys,^9^^,^^10^^,^^18^^–^^20^ our paper questionnaire-based survey is more realistic and suitable for elderly people with poor Internet access in small towns.

One limitation of our research is that we only conducted the survey in Xidian rather than multiple centers. In addition, only vaccinated people were recruited, which might lead to a sample selection bias, although most residents in China are vaccinated. Another limitation is the inadequate evaluation of attitudes and practices, which should be designed via close observation, in-depth interviews, and focus group discussions.

## CONCLUSION

Our findings suggest that most Chinese small-town residents hold a medium level of knowledge of COVID-19 and vaccines, and have optimistic attitudes and proper practices when the COVID-19 epidemic is basically under control. Residents who are > 50 years, have a primary school education or less, and are physical laborers or unemployed have a relatively low knowledge of COVID-19 and vaccines. Females, students, and chronic patients are relatively passive with regard to epidemic control. Our study provides evidence for public health policymakers to identify target populations for health education in the postvaccination era. Because of the limitations in sample representativeness, further studies are required to investigate the KAP of COVID-19 and vaccines among Chinese residents.

## Supplemental files


Supplemental materials
