# Supplementary material for: Knowledge, Attitudes, and Practices toward COVID-19 and Vaccines among Chinese Small-Town Residents: A Cross-sectional Study
Source: Am J Trop Med Hyg. 2022 Jul 25;107(3):551–6. doi: 10.4269/ajtmh.22-0031 (PMC9490646; doi:10.4269/ajtmh.22-0031)
Supplement: Supplementary file 1 [file tpmd220031.SD1.pdf]

Supplemental Table S1. Questionnaire of knowledge, attitudes, and practice toward COVID-19 and vaccines

Questions	Options
**Knowledge** (correct rate, % of the total sample)	
K1.1 The main clinical symptoms of COVID-19 are fever, fatigue, dry cough, and myalgia. (86.9)	True, false, I don't know
K1.2 Unlike the common cold, stuffy nose, runny nose, and sneezing are less common in persons infected with the COVID-19 virus. (30.9)	True, false, I don't know
K1.3 There currently is no effective cure for COVID-19, but early symptomatic and supportive treatment can help most patients recover from the infection. (72.3)	True, false, I don't know
K1.4 Not all persons with COVID-19 will develop to severe cases. Only those who are elderly, have chronic illnesses, and are obese are more likely to be severe cases. (67.7)	True, false, I don't know
K1.5 It is uncertain whether eating or contacting wild animals will result in the infection of COVID-19. (48.9)	True, false, I don't know
K1.6 Patients with COVID-19 cannot infect others when a fever is not present. (67.4)	True, false, I don't know
K1.7 The COVID-19 virus spreads via respiratory droplets from infected individuals. (88.9)	True, false, I don't know
K1.8 Residents can wear masks to prevent the infection by the COVID-19 virus. (97.5)	True, false, I don't know
K1.9 It is not necessary for children and young adults to take measures to prevent the infection by the COVID-19 virus. (93.8)	True, false, I don't know
K1.10 To prevent the infection by COVID-19, individuals should avoid going to crowded places such as train stations and avoid taking public transportations. (98.8)	True, false, I don't know
K1.11 Isolation and treatment of people who are infected with the COVID-19 virus are effective ways to reduce the spread of the virus. (98.0)	True, false, I don't know
K1.12 People who have contact with someone infected with the COVID-19 virus should be immediately isolated in a proper place. In general, the observation period is 14 days. (98.8)	True, false, I don't know
K2.1 The COVID-19 vaccination is legally mandatory. (85.2)	True, false, I don't know
K2.2 The COVID-19 vaccination is indicated in infants <1 years of age. (72.8)	True, false, I don't know
K2.3 The COVID-19 vaccination is indicated in women who are preparing for pregnancy or lactating mothers. (10.9)	True, false, I don't know
K2.4 The COVID-19 vaccination is indicated in patients with acute infection. (82.2)	True, false, I don't know
K2.5 The COVID-19 vaccination is indicated in patients with chronic diseases, such as diabetes, hypertension and heart diseases. (69.4)	True, false, I don't know
K2.6 The COVID-19 vaccination is indicated in person who has already recovered from COVID-19. (56.8)	True, false, I don't know
K2.7 The COVID-19 vaccination is indicated in immunocompromised patients. (30.1)	True, false, I don't know
K2.8 The COVID-19 vaccination is indicated in person allergic to vaccine components. (84.7)	True, false, I don't know
K2.9 For COVID-19 vaccines that require two injections, a better immune effect can be obtained after two injections. (90.6)	True, false, I don't know
K2.10 Mild side effects may occur after COVID-19 vaccination, such as arm pain, redness, fatigue, headache, muscle pain, chills, fever, nausea, etc. (85.9)	True, false, I don't know
K2.11 Other measures (such as wearing a mask and avoiding crowded places) are still important after COVID-19 vaccination. (98.0)	True, false, I don't know
K2.12 Current COVID-19 vaccines are effective in preventing the disease. (94.6)	True, false, I don't know
**Attitudes**	
A1. Do you agree that COVID-19 will finally be successfully controlled?	Agree, disagree, I don't know
A2. Do you have confidence that China can defeat the COVID-19 virus?	Yes, no, I'm not sure
**Practices**	
P1. In recent days, have you gone to any crowded place?	Yes, no
P2. In recent days, have you worn a mask when leaving home?	Yes, no

Supplemental Table S2. Multiple linear regression on factors associated with knowledge of COVID-19 and vaccines

Variable	Coefficient	Standard error	t	P
**Knowledge score of COVID**				
Age group (18-29 vs. 30-49 years)	-0.651	0.219	-2.978	0.003
Age group (50+ vs. 30-49 years)	-0.945	0.211	-4.470	<0.001
Education (primary school and below vs. bachelor’s degree and above)	-1.095	0.210	-5.215	<0.001
Occupation (physical labor vs. mental labor)	-0.633	0.219	-2.892	0.004
Occupation (unemployment vs. mental labor)	-0.831	0.281	-2.958	0.003
**Knowledge score of vaccines**				
Age group (18-29 vs.30-49 years)	-0.673	0.230	-2.930	0.004
Age group (50+ vs.30-49 years)	-0.502	0.215	-2.333	0.020
Education (primary school and below vs. bachelor’s degree and above)	-0.753	0.219	-3.432	0.001
Education (high school vs. bachelor’s degree and above)	-0.420	0.229	-1.833	0.068
**Aggregate knowledge score**				
Age group (18-29 vs. 30-49 years)	-1.355	0.370	-3.665	<0.001
Age group (50+ vs. 30-49 years)	-1.353	0.358	-3.784	<0.001
Education (primary school and below vs. bachelor’s degree and above)	-1.715	0.355	-4.826	<0.001
Occupation (physical labor vs. mental labor)	-0.633	0.370	-1.710	0.088
Occupation (unemployed vs. mental labor)	-1.096	0.475	-2.306	0.022

Supplemental Table S3. Binary logistic regression analysis on factors associated with attitudes toward COVID-19

**Agree on final success (vs disagree or unknow)**	**OR (95%CI)**	**P**
**Gender (female vs. male)**	0.40 (0.18, 0.89)	0.024
**Occupation (students vs. mental labor)**	0.13 (0.03, 0.60)	0.010
**Medical history (yes vs. no)**	0.32 (0.14, 0.76)	0.010
